# Cross-Sectional and Longitudinal Associations between 24-Hour Movement Behaviours, Recreational Screen Use and Psychosocial Health Outcomes in Children: A Compositional Data Analysis Approach

**DOI:** 10.3390/ijerph18115995

**Published:** 2021-06-03

**Authors:** Kar Hau Chong, Anne-Maree Parrish, Dylan P. Cliff, Dorothea Dumuid, Anthony D. Okely

**Affiliations:** 1 Early Start, Faculty of the Arts, Social Sciences and Humanities, University of Wollongong, Wollongong, NSW 2522, Australia; aparrish@uow.edu.au (A.-M.P.); dylanc@uow.edu.au (D.P.C.); tokely@uow.edu.au (A.D.O.); 2 School of Health and Society, Faculty of the Arts, Social Sciences and Humanities, University of Wollongong, Wollongong, NSW 2522, Australia; 3 School of Education, Faculty of the Arts, Social Sciences and Humanities, University of Wollongong, Wollongong, NSW 2522, Australia; 4 Illawarra Health and Medical Research Institute, University of Wollongong, Wollongong, NSW 2522, Australia; 5 Allied Health and Human Performance, Alliance for Research in Exercise, Nutrition and Activity (ARENA), University of South Australia, Adelaide, SA 5001, Australia; Dot.Dumuid@unisa.edu.au

**Keywords:** prospective, activity monitor, accelerometer, mental health, wellbeing, youth, young people, school-aged, observational

## Abstract

It remains unclear whether the time-use composition of 24-h movement behaviours (sleep, sedentary time (ST), physical activity (PA)) and recreational screen use are independently associated with psychosocial health. This study examined the cross-sectional and longitudinal associations between 24-h movement behaviour composition, recreational screen use and psychosocial health outcomes in children. Measures completed at baseline (n = 127; 11.7 years) and follow-up (n = 88; 12.8 years) included accelerometer-based 24-h movement behaviours, self-reported recreational screen use and psychosocial health (Strengths and Difficulties Questionnaire, Kessler’s Psychological Distress Scale). Linear mixed models were used to examine the cross-sectional and longitudinal associations between the 24-h movement behaviour composition and recreational screen use levels with psychosocial health outcomes. Overall, the movement behaviour composition (*p* < 0.05) and recreational screen use levels (*p* < 0.01) were both cross-sectionally but not longitudinally associated with psychosocial health outcomes. Relative to other behaviours, sleep was negatively associated, while light-intensity PA was positively associated with internalising problems and total difficulties scores. ST was positively associated with internalising problems. High levels of recreational screen use (>2 h/day) were associated with greater externalising problems, total difficulties scores and psychological distress. These findings reinforce the importance of achieving a balance between different types of movement behaviours over a 24-h period for psychosocial health.

## 1. Introduction

Psychosocial health problems are increasingly common among children and adolescents, with the worldwide-pooled prevalence of mental disorders estimated to be 13.4% [1]. Anxiety, behavioural and depressive disorders are among the most frequently reported mental health conditions [1,2], accounting for 16% of the global burden of disease and injury among adolescents [3]. Most mental disorders are reported to develop during childhood or adolescence [4], which is likely to have a profound impact on other health and developmental outcomes (e.g., educational underachievement, substance use and abuse) [5]. It is, therefore, important to identify modifiable determinants of psychosocial health for early prevention and intervention.

Physical activity (PA) [6], sedentary behaviour [7], and sleep [8], collectively known as 24-h movement behaviours [9], have been shown to be independently associated with various psychosocial health outcomes (e.g., mental health, anxiety, psychological distress, and behavioural conduct) in children and adolescents. However, because the times spent engaging in these behaviours are mutually exclusive and exhaustive components of a 24-h day (i.e., changing time spent in one behaviour requires compensatory changes in at least one other behaviour), their effects on health should be analysed and interpreted relative to each other rather than in isolation [10,11]. This can be done using compositional data analysis (CoDA), a statistical approach designed to handle compositional data that convey relative information (i.e., only the ratios between the parts of a composition are informative), therefore making it suitable for analysis of time-use data [11,12,13]. To the best of our knowledge, only two cross-sectional studies have examined the association between the full 24-h movement behaviour composition (i.e., time spent in sleep, sedentary time (ST), light-intensity PA (LPA) and moderate- to vigorous-intensity PA (MVPA)) and psychosocial health outcomes in school-aged children and adolescents using a CoDA approach [14,15]. The findings from these studies suggest that overall movement behaviour composition is important for emotional and behavioural development. Given the paucity of research in this area, more studies, particularly of longitudinal design, are needed to better understand the collective and relative associations between 24-h movement behaviours and psychosocial health among children and adolescents. 

There is substantial evidence that high levels of recreational screen use (e.g., television watching, playing computer/video) can have a detrimental effect on children’s and adolescents’ psychosocial health [16,17,18,19]. However, it remains largely unknown whether the effects of screen use are explained by the amount of time spent on screens while sitting (i.e., being sedentary) or the content/context of the screen viewing [18,20]. While it has been proposed that the negative effects of ST may be specific to screen-based activities [20], there remains a lack of evidence justifying whether overall ST (i.e., time spent in all sedentary activities) or specific types of sedentary behaviour are more strongly related to psychosocial health indicators [14]. Most of the published studies on the psychosocial health effects of screen time did not include newer forms of screen technologies (e.g., tablets and smartphones) [16], nor did they account for the confounding influence of time spent in other movement behaviours (i.e., PA and sleep) [16,17,19]. Recreational screen use is a highly prevalent form of sedentary behaviour among children and adolescents worldwide, with a significant proportion (38–76%) not meeting the current recreational screen time recommendations, i.e., no more than 2 h/day [21,22]. In addition, excessive screen use is likely to have a negative impact on other movement behaviours that have also been linked to psychosocial health (e.g., shorter sleep duration) [18,23]. Thus, it is important to determine whether the distribution of time spent in 24-h movement behaviours and recreational screen use contribute independently and uniquely to psychosocial health outcomes. This information has the potential to inform the development of future movement behaviour intervention strategies and guidelines for the promotion of healthy psychosocial development in children and adolescents. 

The purpose of this study was to examine the cross-sectional and longitudinal associations between 24-h movement behaviour composition, recreational screen use and psychosocial health outcomes in a sample of school children. Specifically, this study sought to determine: (1) if the associations between daily movement behaviour composition and psychosocial health outcomes were independent of recreational screen use; and (2) if recreational screen use was associated with psychosocial health outcomes independent of the daily movement behaviour composition. We hypothesised that the daily movement behaviour composition and recreational screen use would be independently associated with children’s psychosocial health outcomes in both cross-sectional and longitudinal analyses.

## 2. Materials and Methods

### 2.1. Study Design and Participants

Data were drawn from a longitudinal study that followed children transitioning from primary school (Year 6; ages 10–12 years) to secondary school (Year 7; ages 11–13 years) [24]. A convenience sample of Government and Independent schools (n = 73) that were located within a 400 km radius of the city of Wollongong in New South Wales, Australia were approached to participate in the study between July 2017 and December 2018. Information sheets and consent forms were sent home with all Year-6 students at the consenting schools to seek parental/legal guardian consent for their participation in the study. At follow-up time points, parents/legal guardians were contacted via a passive consent form (for children who had stayed at the same school) or a new active consent form (for children who had moved to a new school) delivered to their home address or via email to seek their permission for the child to continue participating in the study. Baseline data were collected between April 2018 and August 2019, with follow-ups conducted an average of six months (range 4–9 months) (Follow-up 1: October 2018–March 2020) and 12 months (range 11–16 months) (Follow-up 2: April 2019–November 2019) after baseline using a standardised data collection protocol (i.e., 24-h accelerometer wearing, completing a questionnaire and anthropometric measurements). The study was terminated in April 2020 as the University officially suspended all face-to-face data collection due to COVID-19. 

The current study analysed data collected at two time points. For the cross-sectional analysis, the analytical sample included participants who had valid accelerometry, recreational screen use and psychosocial health data at baseline (Year 6; ages 10–12 years; referred to hereafter as T1). For the longitudinal analysis, the analytical sample comprised of participants who had valid accelerometry and recreational screen use data at T1 and psychosocial health data at one follow-up time point during the secondary school period (Year 7; ages 11–13 years; referred to hereafter as T2). Due to the variation at the baseline time frames between schools, some participants had only one data point for T2, while others had two data points for T2. In the latter cases, the data point with the longest follow-up time was included to strengthen the robustness of the longitudinal results.

#### Ethical Approval

The conduct of this study was reviewed and approved by the Human Research Ethics Committee of the University of Wollongong (2017/255) and the New South Wales Department of Education (SERAP No. 2018365).

### 2.2. Measures

#### 2.2.1. 24-h Movement Behaviours

Time spent in sleep, ST, LPA and MVPA was assessed using a wrist-worn GENEActiv accelerometry (ActivInsights Ltd., Cambridgeshire, UK) on the non-dominant wrist for six consecutive days (24 h/day). The GENEActiv accelerometer has been validated for the assessment of PA [25] and ST [26] in children. Accelerometer data (sampled at 75 Hz) were downloaded using GENEActiv PC software version 3.2 (ActivInsights Ltd., Cambridgeshire, UK) and saved in raw format as binary files. The data files were then processed in R using the GGIR package (version 1.10–7) [27], which auto-calibrated the raw triaxial accelerometer signals [28] and computed the Euclidean Norm Minus One (ENMO) metric (i.e., gravity-corrected vector magnitude units) [29]. ENMO values were averaged over 5 s epochs and expressed in milli-gravitational units (m*g*). Accelerometer non-wear time was estimated based on the standard deviation and value range of each accelerometer axis, calculated over 60 min windows with 15 min increments [29]. For each 15 min period detected as non-wear time over the valid wearing days, missing data were imputed using the mean values calculated from valid data at the same time points on other days [27]. A sleep detection algorithm (based on the distribution of change in arm angle) developed by van Hees and colleagues [30] was applied to identify the sleep period time window for the estimation of sleep duration (including awakening periods). This algorithm has been applied previously in wrist-worn accelerometry studies involving children [14,31,32]. Waking time data were further categorised as ST (ENMO < 52 m*g*), LPA (ENMO 52–191 m*g*) or MVPA (ENMO ≥ 192 m*g*) using validated acceleration intensity thresholds [33,34]. To be included in the analysis, participants were required to have at least three valid days of accelerometer data (i.e., at least 16 h of wear time per day; including one weekend day) [32]. Any days with ≤200 min of sleep duration or ≥1000 min of ST were considered invalid and were excluded from the analysis [35]. The average time spent in each behaviour was weighted at 5:2 for weekdays and weekend days. 

#### 2.2.2. Recreational Screen Use

Participants were asked to report the amount of time spent engaging in a range of sedentary and screen-based activities (i.e., while sitting) on each weekday (during out-of-school hours only) and weekend day during a typical school week. These activities were adapted from the revised version of the Adolescent Sedentary Activity Questionnaire (ASAQ) [36]; with an additional item assessing the use of screen devices for social purposes (e.g., text/instant messaging, using social networking sites) to reflect current trends in children’s screen media use. In the current study, daily recreational screen time was calculated by taking a weighted average of total time spent in five screen-based sedentary activities (i.e., watching television, using a computer/laptop for entertainment, using a smartphone/tablet for entertainment, playing computer/video games and using screen devices for social purposes) (Table S1) on weekdays and weekend days: (average of weekdays*5 + average of weekends*2)/7. For analysis purposes, participants were classified into low (<=2 h/day) or high (>2 h/day) levels of recreational screen use based on the Australian sedentary recreational screen time recommendation [37].

#### 2.2.3. Psychosocial Health Measures

Psychosocial health were assessed using the self-report version of the Strengths and Difficulties Questionnaire (SDQ) [38] and the Kessler’s Psychological Distress Scale (K-10) [39]. These questionnaires have been used in the Australian population surveys [17,40], indicating the appropriateness and acceptability of the questionnaires among Australian children and adolescents.

The SDQ [38] consists of 25 items equally divided across five subscales assessing emotional and behavioural problems in children: emotional problems, conduct problems, hyperactivity, peer problems and prosocial behaviours. It has demonstrated acceptable construct validity [41] and sensitivity to detect change in mental health functioning among children and adolescents [42], making it appropriate for longitudinal studies. In this study, the self-report version of the SDQ was used where participants were asked to indicate the degree to which each item applies to them over the last six months using a 3-point Likert scale: ‘not true’, ‘somewhat true’, or ‘certainly true’. ‘Somewhat true’ is always scored as 1, but the scoring of ‘not true’ and ‘certainly true’ varies across items (0 or 2). The scores for each item were calculated and subsequently used to compute three subscale scores for assessing internalising problems (the sum of the emotional problems and peer problems subscales; range 0–20), externalising problems (the sum of the conduct problems and hyperactivity subscales; range 0–20), and prosocial behaviour (range 0–10). This three-subscale SDQ model has been recommended for use in studies involving low-risk or general population samples [43]. In the current study, the internal consistency (Cronbach’s alpha, α) for the three subscales (internalising problems = 0.69; externalising problems = 0.74; prosocial behaviours = 0.64) were acceptable. A total difficulties score was also computed by summing the scores of the internalising problems and externalising problems subscale (range 0–40). The calculated scores for the four main outcome variables (internalising problems, externalising problems, total difficulties scores and prosocial behaviour) were further categorised as ‘close to average’, ‘slightly raised’, ‘high’ or ‘very high’ based on the recommended four-band classification [44]. 

The K-10 scale [39] is a 10-item self-report questionnaire developed for assessing non-specific psychological distress based on the level of anxiety and depressive symptoms experienced during the past 4-week period. Participants were asked to indicate the degree to which each item applied to them using a 5-point Likert scale (1 = ‘none of the time’, 2 = ‘a little of the time’, 3 = ‘some of the time’, 4 = ‘most of the time’, 5 = ‘all of the time’). A total score was calculated by summing the scores of all 10 items (range 10–50), with higher scores representing higher levels of psychological distress. The calculated scores were further categorised as ‘low’, ‘moderate’, ‘high’ or ‘very high’ using the recommended cut-points [45]. The K-10 scale has demonstrated sufficient unidimensionality [46] and acceptable internal consistency (Cronbach’s alpha, α = 0.70–0.90) [40,47] among Australian children and adolescents aged 11–17 years. In the current study, the internal consistency of this scale was high (α = 0.84).

#### 2.2.4. Control Variables

The following variables were included as covariates in the analyses: socio-demographic characteristics (age, sex and socio-economic status), body mass index z-score (BAZ), pubertal progression and experience with transition from primary to secondary school. These variables were selected due to their associations with movement behaviours [48] and/or psychosocial health outcomes [49,50] in children and adolescents. 

Socio-demographic information (sex, date of birth and home postcode) was provided by the parents/legal guardians on the consent form. A standardised measure of socio-economic status (Socio-Economic Indexes for Areas Index of Relative Socio-Economic Disadvantage; SEIFA IRSD) [51] was derived from the home postcode provided, with higher values indicating relatively less disadvantaged. Body weight was measured using a digital scale (Model 874; SECA, Hamburg, Germany), and height was measured using a portable stadiometer (Model 217; SECA, Hamburg, Germany). BAZ was calculated using AnthroPlus software version 1.0.4 (World Health Organization, Geneva, Switzerland) based on the 2007 World Health Organization growth reference [52]. Pubertal progression was assessed using an average score derived from three general (i.e., growth spurt in height, body hair growth, skin change) and two sex-specific (boys: facial hair growth and voice change; girls: breast development and the onset of menarche) indicators of puberty [53]. Each indicator was scored on a 4-point Likert scale from 1 ‘has not started yet’ (or ‘no’ for menarche item) to 4 ‘seems completed’ (or ‘yes’ for menarche item). A change in pubertal progression score was also computed (T2 estimates minus T1 estimates) for use in the longitudinal analyses. At T2, participants were asked to rate their experiences in transitioning to secondary school using a four-level Likert scale: ‘difficult’, ‘somewhat difficult’, ‘somewhat easy’ or ‘easy’ (adapted from Waters et al. [50]) These data were further categorised as having a difficult (combined ‘difficult’ and ‘somewhat difficult’ responses) or easy (combined ‘somewhat easy’ and ‘easy’ responses) transition experience and were included in the longitudinal analyses. 

### 2.3. Statistical Analyses

Standard statistical analyses were performed in IBM SPSS Statistics for Windows (version 26; IBM Corp., Armonk, NY, USA). Descriptive statistics (mean, standard deviation, frequency and percentage) were calculated to summarise participant characteristics. Differences between participants included and excluded from the cross-sectional or longitudinal analyses were analysed using independent t-tests. Changes in participant characteristics from T1 to T2 were analysed using paired sample t-tests or McNemar-Bowker’s tests.

Compositional data analyses were performed in R (version 3.6.2; R Foundation for Statistical Computing, Vienna, Austria) using the ‘compositions’ (version 2.0.0) [54], ‘lme4′ (version 1.1–23) [55] and ‘lmerTest’ (version 3.1–3) [56] packages. Compositional descriptive statistics, including compositional mean (measure of central tendency) and pairwise log-ratio variation matrix (measure of dispersion/variability), were used to describe the daily composition of 24-h movement behaviours [11]. The four-part movement behaviour composition was expressed as four specific sets of three isometric log-ratio (ilr) coordinates for statistical analyses, each of which included a different behaviour (either sleep, ST, LPA or MVPA) relative to all remaining behaviours as the first ilr [11,13]. Association analyses were conducted using the linear mixed models to account for the random effect of school clustering (school site as random intercept). For cross-sectional analyses, four models were constructed for each T1 psychosocial health outcome (as the dependent variable); each model included one of the four sets of T1 movement behaviour ilrs created and recreational screen use (low vs. high levels) as the explanatory variables. This was done so that each model analysed the association of one specific behaviour relative to all remaining behaviours. All cross-sectional models were adjusted for socio-demographic characteristics, BAZ and pubertal progression score. The longitudinal analyses were conducted using a similar approach by including T2 psychosocial health outcome as the dependent variable, and the sets of T1 movement behaviour ilrs and recreational screen use as the explanatory variables. All longitudinal models were adjusted for socio-demographic characteristics, BAZ (T1), change in pubertal progression score, school transition experiences and the corresponding T1 psychosocial health outcome. All models were checked for linearity, normality and homoscedasticity of residuals, and outliers to ensure assumptions were not violated.

The ANOVA table of the model fit was checked for the statistical significance of the associations between the set of movement behaviour ilrs (representing the daily movement behaviour composition) and recreational screen use with each psychosocial health outcome. If a significant association (*p* < 0.05) was observed for the set of movement behaviour ilrs, the regression coefficients and statistical significance of the first ilr from the four models were examined to determine the health association of each behaviour relative to all remaining behaviours. 

## 3. Results

**Figure 1:**
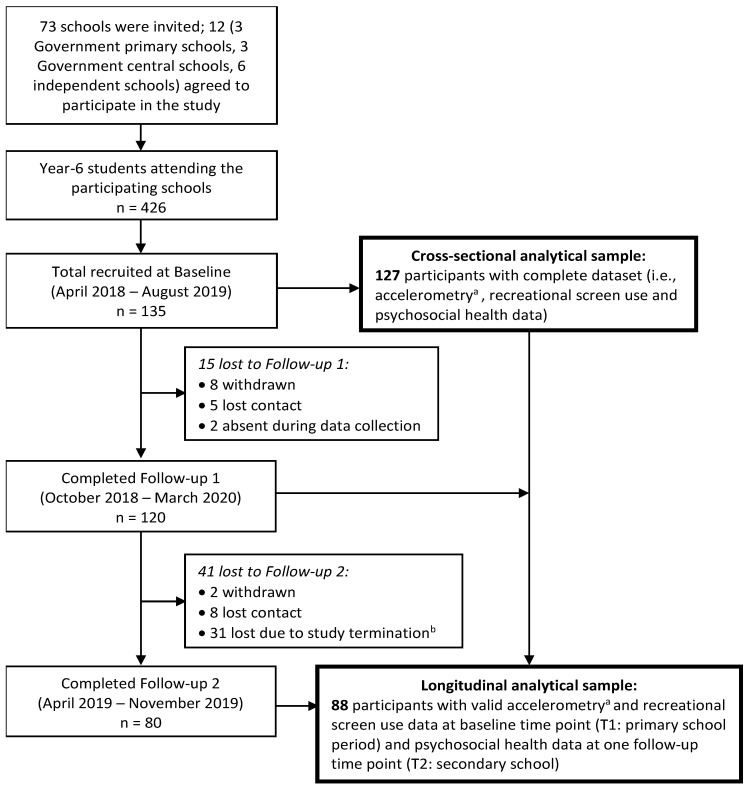
Flow chart of participants through the study. *Note*. ^a^ Provided at least three valid days of accelerometer data (i.e., at least 16 h of wear time per day, including one weekend day); ^b^ Study was terminated in April 2020 as the University officially suspended all face-to-face data collection due to COVID-19.

A total of 426 Year-6 children from 12 participating schools were invited to participate in this study, of which 135 (59 boys, 76 girls; mean age = 11.7 ± 0.5 years) (response rate = 31.7%) provided written parental consent and verbal assent and completed the study protocol at T1 (Figure 1). Of these, 121 (89.6%) completed at least one of the follow-up assessments. Eight participants did not provide valid accelerometry data at T1, leaving a sample size of 127 (94.1%) for the cross-sectional analysis. Of this sample, 88 (69.3%) also provided psychosocial health data at T2 (mean follow-up duration = 12 ± 2 months) and were included in the longitudinal analysis. The longitudinal analytical sample was older (*p* = 0.015) and of a higher socio-economic status (*p* < 0.0001) than the drop-out sample (Table S2). 

The descriptive characteristics of the cross-sectional and longitudinal samples are summarised in Table 1. The average daily movement behaviour compositions were similar between the two samples at T1, with the largest proportion of 24-h period spent in ST (42%), followed by sleep (38%), LPA (15%) and then MVPA (5%). Most participants (>60%) spent more than two hours per day on recreational screen use. The pairwise log-ratio variances indicated that sleep and ST had the highest co-dependence, whereas MVPA had the lowest co-dependence with the other three behaviours in both cross-sectional and longitudinal samples (Table S3). More than half the participants had their SDQ subscale (internalising problems, externalising problems, prosocial behaviour) and total scores (total difficulties scores) in the ‘close to average’ range, and had low/moderate levels of psychological distress, with no significant differences observed in the proportions between T1 and T2 (Table S4). Of the longitudinal sample, 4.5% (n = 4) had changed schools since T1 and 36.4% (n = 32) were reported to have experienced a difficult transition to secondary school.

Table 2 presents the compositional regression models for the cross-sectional association analyses. The daily movement behaviour composition was significantly associated with internalising problems (*p* = 0.021) and total difficulties scores (*p* = 0.030), but not with other outcomes, after adjusting for recreational screen use levels and other covariates. Time spent in sleep relative to the other behaviours was negatively associated with internalising problems (β*_ilr_* = −6.85, *p* = 0.017) and total difficulties scores (β*_ilr_* = −13.31, *p* = 0.006). Time spent in ST relative to the other behaviours was positively associated with internalising problems (β*_ilr_* = 4.19, *p* = 0.046). Time spent in LPA relative to the other behaviours was also positively associated with internalising problems (β*_ilr_* = 4.18, *p* = 0.044) and total difficulties scores (β*_ilr_* = 7.54, *p* = 0.031). These models further revealed that recreational screen use levels were significantly associated with psychosocial health outcomes, independent of the movement behaviour composition. Children with a high level of screen use (>2 h/day) had greater externalising problems (β = 2.66, *p* < 0.0001), total difficulties scores (β = 3.68, *p* = 0.001) and psychological distress (β = 3.69, *p* = 0.005) compared to those with a low level of screen use (≤2 h/day).

Table 3 presents the compositional regression models for the longitudinal association analyses. There were no significant associations between movement behaviour composition (*p* = 0.08–0.56) or recreational screen use levels (*p* = 0.08–0.70) at T1 with any of the psychosocial health outcomes at T2. 

## 4. Discussion

This study investigated the cross-sectional and longitudinal associations between the 24-h movement behaviour composition and recreational screen use with psychosocial health outcomes in children. Overall, the cross-sectional analyses revealed that the daily movement behaviour composition and recreational screen use levels were independently associated with children’s psychosocial health outcomes. The movement behaviour composition was significantly associated with internalising problems and total difficulties scores. In particular, more time spent in sleep and less time spent in LPA (relative to other behaviours) were associated with less internalising problems and total difficulties scores. Conversely, more time spent in ST relative to other behaviours was associated with greater internalising problems. High levels of recreational screen use (>2 h/day) were significantly associated with greater externalising problems, total difficulties scores and psychological distress. In the longitudinal analyses, however, no significant associations were observed between the movement behaviour composition or recreational screen use levels and any of the psychosocial health outcomes. 

Our cross-sectional findings confirm and extend previous CoDA research [14,15] by showing that the association between daily movement behaviour composition and psychosocial health outcomes is independent of children’s recreational screen use levels. These findings align with the 24-h movement guidelines [9,37,57], which recommend a daily balance of time spent in various types of movement behaviours for optimal health. Consistent with the findings of Fairclough et al. [14], we found that the movement behaviour composition was associated with internalising (emotional and peer/relationship problems) but not externalising problems (conduct problems and hyperactivity). However, contrary to Fairclough et al.’s study [14], there was no association between movement behaviour composition and prosocial behaviour in the current study. This could be because a large proportion of the sample had a ‘normal’ prosocial behaviour profile (i.e., 74% had prosocial scores in the ‘close to average’ range), which may have limited the precision and ability to detect an association between movement behaviour composition and this outcome variable (i.e., a ceiling effect) [14,21]. Another potential explanation is that the types or contexts of activities performed (e.g., team sports or community-based activities) may also play an important role in fostering prosocial development [58,59], which could not be determined from the accelerometer-based time-use data. Nonetheless, we found no evidence of a longitudinal association between the movement behaviour composition and any of the assessed health outcomes. This may be due to the small sample size and limited changes in the cohort’s psychosocial health profiles over the follow-up time period. Additional research with a larger, more representative sample and longer follow-up periods is warranted to confirm this finding.

Longer duration in sleep relative to other behaviours was cross-sectionally associated with less internalising problems and total difficulties scores in the present study. This finding is consistent with existing literature that shows favourable associations between sleep duration and emotional and behavioural problems in the school-aged population [8,15]. This is concerning given the declining sleep duration trajectories observed during childhood and adolescence [60,61], with the global proportion of children meeting the current sleep duration recommendations (i.e., 9–11 h/night for ages 5–13 years) ranging from 18% to 76% (based on accelerometer measures) [22]. There is also considerable evidence that meeting the sleep duration recommendations is more strongly and consistently associated with better mental health outcomes than that observed with meeting the screen time or PA recommendations within the 24-h movement guidelines [62,63,64]. This further highlights the critical role of sleep in promoting positive psychosocial development and should therefore be prioritised in future movement behaviour change interventions. 

We found that higher ST (relative to other behaviours) was cross-sectionally associated with greater internalising problems, which is consistent with the findings of Fairclough et al. [14]. Because its compositional association with internalising problems was observed to be independent of children’s recreational screen use levels, our results suggest that the negative association between ST and psychosocial health may not be entirely attributable to screen-based activities. Another notable finding of the current study is that high levels of recreational screen use (>2 h/day) were cross-sectionally and negatively associated with externalising problems and psychological distress independent of the movement behaviour compositions. This implies that excessive screen-based sedentary activities during recreational time may be a distinct risk factor for psychosocial health [7,19,65]. Despite the lack of observed longitudinal associations, our findings do provide support for the current movement guidelines of limiting the daily amount of ST, including no more than 2 h of recreational screen use [9,37,57,66] for psychosocial health benefits in the school-aged population. 

We found that more time spent in LPA relative to other behaviours was associated with greater internalising problems and total difficulties scores. This finding is in line with other CoDA studies [14,15,67] that have also found a negative association between LPA and various health and developmental outcomes (e.g., higher adiposity and blood pressure, poorer executive function and academic achievement) among children and adolescents. This may be explained by the displacement of time spent in the remaining movement behaviours [67]; whereby an increase in LPA will require an equal decrease in time spent in at least one other behaviour within the 24-h period due to the compositional nature of time-use data. This means that children who spent more time in LPA would have less time available for other movement behaviours (e.g., sleep) that may foster positive psychosocial development. In other words, the negative association of LPA with psychosocial health could be due the displacement effect rather than the time spent in LPA per se. 

Consistent with other CoDA studies [14,15], we found no significant association between time spent in MVPA (relative to other behaviours) and psychosocial health. The null association does not imply that MVPA per se is not related to psychosocial development; but rather that the amount of time spent in MVPA in association with the trade-offs in time from the other movement behaviours that the children had to make is neutral in relation to psychosocial health [67]. In fact, recent research that has applied compositional isotemporal substitution models demonstrates that reallocations of time between movement behaviours may have important implications for health in children and adolescents. Specifically, reallocating time from MVPA to the other behaviours (especially ST) was consistently associated with greater and unfavourable predicted differences in various health outcomes (e.g., adiposity, fitness, internalising difficulties) than the reverse [14,15,32,68]. Together, these findings emphasise the importance of considering the overall 24-h movement behaviour composition when examining and/or interpreting the health associations of MVPA.

Our study found differential health associations for the overall movement behaviour composition (i.e., associated with internalising problems only) and recreational screen use (i.e., associated with externalising problems and psychological distress), which suggests that different types of behaviours may contribute to different dimensions of psychosocial health. This underscores the importance of considering the influences of both intensity- and domain-specific movement behaviours when determining the ‘ideal’ daily time-use composition [69] for optimal psychosocial health. Further research is needed to investigate the directionality and potential mechanisms underlying the compositional associations between movement behaviours and psychosocial health.

To our knowledge, this is the first compositional analysis study to investigate the independent associations of 24-h movement behaviour composition and recreational screen use levels with children’s psychosocial health outcomes in both cross-sectional and longitudinal settings. Strengths of this study included accelerometer-based measures of 24-h movement behaviours and the use of an appropriate analytical approach to account for the compositional nature of time-use data. The statistical models were also adjusted for the important covariates (e.g., puberty) that might confound the associations of interests during this particular developmental period. A limitation of this study is the small sample size, which may limit the generalisability of the study findings. We were also unable to perform a statistical power calculation in this study due to the lack of post hoc analysis methods available for compositional linear mixed models. Next, as with all self-report measures, the self-reported data on psychosocial health and recreational screen use are subject to social desirability and recall bias. It should also be noted that the self-report measure of recreational screen use was not time matched with the accelerometer data (i.e., not being assessed concurrently for the same time frame); and the dichotomisation of screen use may have underestimated the extent of variation in outcomes between groups. Thus, caution is warranted in interpreting these results. Because this study focused mainly on the volume and intensity of the behaviours, it remains unclear whether the observed associations with psychosocial health are moderated or mediated by the other aspects of the behaviours (e.g., types or contexts of PA, content of screen time). Lastly, we acknowledge that the use of intensity cut-points, while validated, may have resulted in some misclassification of movement behaviours as they do not account for upper limb movements during sedentary or stationary light-intensity activities [70]. 

## 5. Conclusions

This study found that the 24-h movement behaviour composition and recreational screen use were independently associated with children’s psychosocial health, although these associations were only evident in cross-sectional but not longitudinal analyses. Specifically, within a 24-h context, spending more time in sleep and less time in ST and LPA while engaging in low levels of recreational screen activities may be beneficial for psychosocial health in children. These findings reinforce the importance of achieving a balance between different types of movement behaviours over a 24-h period for optimal health benefits.

## Figures and Tables

**Table 1:** Descriptive characteristics of the participants.

Characteristics	Cross-Sectional Sample (n = 127)	Longitudinal Sample (n = 88)		
		T1	T2	*p*-Value for Change ^a^
Age (years)	11.7 (0.5)	11.8 (0.4)	12.8 (0.4)	
Sex, n (%) girls	73 (57.5)	52 (59.1)	-	
Socio-economic status (SEIFA IRSD)	987 (62)	1003 (44)	-	
BAZ ^b^	0.44 (1.14)	0.41 (1.12)	0.44 (1.14)	0.44
Pubertal progression score	2.02 (0.55)	2.03 (0.55)	2.41 (0.64)	<0.0001
Psychosocial health				
Internalising problems	5.5 (3.4)	5.4 (3.4)	5.5 (3.7)	0.91
Externalising problems	6.6 (3.3)	6.7 (3.2)	6.6 (3.3)	0.90
Total difficulties scores	12.1 (5.8)	12.2 (5.7)	12.1 (5.7)	1.00
Prosocial behaviour	7.8 (1.8)	7.9 (1.9)	7.8 (1.6)	0.56
Psychological distress	21.5 (6.8)	21.2 (6.7)	21.7 (7.5)	0.43
Movement behaviours composition, mins/day ^c,d^ (% of 24-h)				
Sleep	544 (37.8)	548 (38.0)	-	
ST	614 (42.6)	607 (42.2)	-	
LPA	213 (14.8)	217 (15.1)	-	
MVPA	69 (4.8)	68 (4.7)	-	
Recreational screen use, mins/day ^d^	207 (131)	189 (124)	-	
High screen use (>2 h/day), n (%) ^d^	86 (67.7)	56 (63.6)	-	

Data presented as mean (standard deviation) unless indicated. *SEIFA* Socio-economic Indicators for Areas, *IRSD* Index of Relative Social Disadvantage, *BAZ* body mass index z-score, *ST* sedentary time, *LPA* light-intensity physical activity, *MVPA* moderate- to vigorous-intensity physical activity. ^a^ Examined using paired sample *t*-test; ^b^ Missing data for one participant at T2; ^c^ Presented as compositional means, adjusted to 1440 min (24 h); ^d^ Data at T2 were not reported as they were out-of-scope for this study.

**Table 2:** Cross-sectional associations between 24-h movement behaviour composition, recreational screen use levels and psychosocial health outcomes (n = 127).

Psychosocial Health Outcomes (T1)	Movement Behaviours Composition (T1)										Recreational Screen Use levels (T1) ^a^		
	Overall Composition		Sleep		ST		LPA		MVPA				
	*X* ^2^	*p*	β*_ilr_*	*p*	β*_ilr_*	*p*	β*_ilr_*	*p*	β*_ilr_*	*p*	*X* ^2^	β	*p*
Internalising problems	**9.70**	**0.021**	**−6.85**	**0.017**	**4.19**	**0.046**	**4.18**	**0.044**	−1.53	0.19	2.01	0.97	0.16
Externalising problems	6.84	0.08	−6.34	0.02	2.40	0.22	3.22	0.10	0.72	0.51	**17.23**	**2.66**	**<0.0001**
Total difficulties	**8.93**	**0.030**	**−13.31**	**0.006**	6.48	0.06	**7.54**	**0.031**	−0.72	0.71	**10.54**	**3.68**	**0.001**
Prosocial behaviour	5.03	0.17	1.11	0.47	−1.38	0.23	4.61	0.25	0.89	0.16	0.42	−0.24	0.52
Psychological distress	4.24	0.24	−10.34	0.05	4.96	0.21	5.67	0.15	−0.29	0.89	**7.98**	**3.69**	**0.005**

β*_ilr_* represents isometric log-ratio regression coefficient of the specific movement behaviour relative to all remaining behaviours. *ST* sedentary time, *LPA* light-intensity physical activity, *MVPA* moderate- to vigorous-intensity physical activity. All models are adjusted for socio-demographic characteristics (age, sex, socio-economic status), body mass index z-score, pubertal progression score and school clustering. Values in bold indicate statistically significant associations (*p* < 0.05). ^a^ Low level of recreational screen use (i.e., ≤2 h/day) as the reference category.

**Table 3:** Longitudinal associations between 24-h movement behaviour composition, recreational screen use levels and psychosocial health outcomes (n = 88).

Psychosocial Health Outcomes (T2)	Movement Behaviours Composition (T1)										Recreational Screen Use Levels (T2) ^a^		
	Overall Composition		Sleep		ST		LPA		MVPA				
	*X* ^2^	*p*	β*_ilr_*	*p*	β*_ilr_*	*p*	β*_ilr_*	*p*	β*_ilr_*	*p*	*X* ^2^	β	*p*
Internalising problems	4.75	0.19	3.52	0.23	−0.50	0.83	−1.37	0.53	−1.65	0.15	1.54	0.79	0.21
Externalising problems	2.04	0.56	3.48	0.22	−2.24	0.32	−0.08	0.97	−1.16	0.28	1.61	0.84	0.21
Total difficulties	4.49	0.21	6.78	0.13	−2.84	0.42	−1.27	0.70	−2.67	0.11	2.79	1.68	0.09
Prosocial behaviour	6.84	0.08	−0.45	0.74	−1.35	0.22	1.41	0.16	0.40	0.45	0.15	0.12	0.70
Psychological distress	4.89	0.18	5.72	0.36	1.58	0.76	−4.48	0.35	−2.82	0.26	3.13	2.54	0.08

*β_ilr_* represents isometric log-ratio regression coefficient of the specific movement behaviour relative to all remaining behaviours. *ST* sedentary time, *LPA* light-intensity physical activity, *MVPA* moderate- to vigorous-intensity physical activity. All models are adjusted for socio-demographic characteristics (T1 age, sex, socio-economic status), change in pubertal progression score, school transition experiences, T1 measures (body mass index z-score and corresponding psychosocial health outcome) and school clustering. ^a^ Low level of recreational screen use (i.e., ≤2 h/day) as the reference category.

## Data Availability

The data presented in this study are not openly available as participants did not provide informed consent for data sharing.

## Supplementary Materials

The following are available online at https://www.mdpi.com/article/10.3390/ijerph18115995/s1, Table S1: List of survey items used to assess sedentary recreational screen use, Table S2: Descriptive characteristics (Time 1) of the participants included and excluded from longitudinal analysis, Table S3: Pairwise log-ratio variation matrix for sleep, sedentary time, light-intensity physical activity and moderate- to vigorous-intensity physical activity at baseline (T1), Table S4: Classifications of psychosocial health status, n (%).
