# Supplementary material for: Cross-Sectional and Longitudinal Associations between 24-Hour Movement Behaviours, Recreational Screen Use and Psychosocial Health Outcomes in Children: A Compositional Data Analysis Approach
Source: Int J Environ Res Public Health. 2021 Jun 3;18(11):5995. doi: 10.3390/ijerph18115995 (PMC8199728; doi:10.3390/ijerph18115995)
Supplement: Supplementary file 1 [file ijerph-18-05995-s001.zip › ijerph-1238114-supplementary.pdf]

Supplementary Material

Table S1. List of survey items used to assess sedentary recreational screen use.

	Activity Items Included in the Calculation of Sedentary Recreational Screen Time
1.	Watching TV (including videos/DVDs)
2.	Using a computer/laptop for entertainment (e.g. watching videos/movies/YouTube, listening to music, surfing the net)
3.	Using a smartphone/tablet (e.g. iPad) for entertainment (e.g. watching videos/YouTube, listening to music, surfing the net)
4.	Playing computer or video games (Nintendo, Xbox, PlayStation, Wii)
5.	Using any screen devices for social purposes (e.g. text/instant messaging, Facebook, Instagram, Snapchat, Tumblr, Twitter)

Table S2. Descriptive characteristics (Time 1) of the participants included and excluded from longitudinal analysis.

Characteristics	Included Sample (n = 88)	Excluded Sample (n = 39)	*p*-value [a]
Age (years)	11.8 (0.4)	11.5 (0.6)	0.015
Sex, % girls	59.1	53.8	0.58
Socio-economic status (SEIFA IRSD)	1003 (44)	950 (79)	<0.0001
BAZ	0.41 (1.12)	0.50 (1.18)	0.67
Pubertal progression score	2.03 (0.55)	1.98 (0.55)	0.58
Psychosocial health			
Internalising difficulties	5.4 (3.4)	5.7 (3.5)	0.65
Externalising difficulties	6.7 (3.2)	6.3 (3.3)	0.48
Total difficulties	12.2 (5.7)	12.0 (6.1)	0.90
Prosocial behaviour	7.9 (1.9)	7.5 (1.7)	0.21
Psychological distress	21.2 (6.7)	22.2 (7.0)	0.45

Data presented as mean (standard deviation) unless indicated. *SEIFA* Socio-economic Indicators for Areas, *IRSD* Index of Relative Social Disadvantage, *BAZ* body mass index z-score. [a] Differences between samples were tested using independent t-tests.

Table S3. Pairwise log-ratio variation matrix for sleep, sedentary time, light-intensity physical activity and moderate- to vigorous-intensity physical activity at baseline (T1).

Movement	Cross-Sectional Sample (n=127)				Longitudinal Sample (n=88)			
behaviour	Sleep	ST	LPA	MVPA	Sleep	ST	LPA	MVPA
Sleep	0	0.010	0.014	0.117	0	0.029	0.038	0.149
ST	0.010	0	0.011	0.109	0.029	0	0.062	0.224
LPA	0.014	0.011	0	0.129	0.038	0.062	0	0.126
MVPA	0.117	0.109	0.129	0	0.149	0.224	0.126	0

Note. Value ranges between 0 and 1, with a value closer to zero indicating higher co-dependency between the two behaviours. *ST* sedentary time, *LPA* light-intensity physical activity, *MVPA* moderate- to vigorous-intensity physical activity.

Table S4. Classifications of psychosocial health status, n (%).

Classifications [a]	Cross-Sectional Sample (n = 127)	Longitudinal Sample (n = 88)		
		T1	T2	*p*-value for difference [b]
Internalising Problems				
Close to average (0–6)	82 (64.6)	58 (65.9)	59 (67.0)	0.77
Slightly raised (7–8)	18 (14.2)	14 (15.9)	9 (10.2)	
High (9)	5 (3.9)	3 (3.4)	8 (9.1)	
Very high (10–20)	22 (17.3)	13 (14.8)	12 (13.6)	
Externalising problems				
Close to average (0–8)	91 (71.7)	64 (72.7)	61 (69.3)	0.86

Slightly raised (9–10)	20 (15.7)	12 (13.6)	17 (19.3)	
High (11–12)	10 (7.9)	6 (6.8)	6 (6.8)	
Very high (13–20)	6 (4.7)	6 (6.8)	4 (4.5)	
Total difficulties scores				
Close to average (0–14)	86 (67.7)	62 (70.5)	59 (67.0)	0.76
Slightly raised (15–17)	16 (12.6)	10 (11.4)	11 (12.5)	
High (18–19)	7 (5.5)	5 (5.7)	7 (8.0)	
Very high (20–40)	18 (14.2)	11 (12.5)	11 (12.5)	
Prosocial behaviour				
Close to average (7–10)	94 (74.0)	66 (75.0)	69 (78.4)	0.68
Slightly raised (6)	16 (12.6)	11 (12.5)	8 (9.1)	
High (5)	10 (7.9)	7 (8.0)	9 (10.2)	
Very high (0–4)	7 (5.5)	4 (4.5)	2 (2.3)	
Psychological distress				
Low (10–15)	21 (16.5)	15 (17.0)	21 (23.9)	0.55
Moderate (16–21)	54 (42.5)	39 (44.3)	30 (34.1)	
High (22–29)	32 (25.2)	23 (26.1)	23 (26.1)	
Very high (30–50)	20 (15.7)	11 (12.5)	14 (15.9)	

[a] The recommended score range for each classification category is presented in brackets. [b] Examined using McNemar-Bowker's test.
